# Serotonin 2A Receptor SNP rs7330461 Association with Treatment Response to Pomaglumetad Methionil in Patients with Schizophrenia

**DOI:** 10.3390/jpm6010009

**Published:** 2016-02-05

**Authors:** Laura K. Nisenbaum, AnnCatherine M. Downing, Fangyi Zhao, Brian A. Millen, Leanne Munsie, Bruce J. Kinon, David H. Adams, Juan Carlos Gomez, Michelle Ann Penny

**Affiliations:** 1 Eli Lilly and Company, Indianapolis, IN 46285, USA; downing_anncatherine_m@lilly.com (A.M.D.); zhao_fangyi@lilly.com (F.Z.); millen_brian_a@lilly.com (B.A.M.); munsie_leanne_m@lilly.com (L.M.); adams_david_henry@lilly.com (D.H.A.); gomez_juan-carlos@lilly.com (J.C.G.); 2 Lundbeck LLC, Four Parkway North, Deerfield, IL 60015, USA; BKIN@lundbeck.com; 3 Computational Biology and Genomics, Biogen, 225 Binney Street, Cambridge, MA 02142, USA; michelle.penny@biogen.com

**Keywords:** schizophrenia, *HTR2A*, pomaglumetad methionil

## Abstract

This study aims to confirm the initial pharmacogenetic finding observed within the clinical proof-of-concept trial of an enhanced response to treatment with pomaglumetad methionil (LY2140023 monohydrate) in Caucasian schizophrenia patients homozygous for T/T at single nucleotide polymorphism rs7330461 in the serotonin (5-hydroxytryptamine) 2A receptor gene compared to A/A homozygous patients. The effect of the rs7330461 genotype on the response to pomaglumetad methionil treatment was assessed in three additional clinical trials and in an integrated analysis. Overall, this study includes data from 1115 Caucasian patients for whom genotyping information for rs7330461 was available, consisting of 513 A/A homozygous, 466 A/T heterozygous and 136 T/T homozygous patients. Caucasian T/T homozygous patients showed significantly (*p* ≤ 0.05) greater improvement in Positive and Negative Syndrome Scale (PANSS) total scores during treatment with pomaglumetad methionil 40 mg twice daily compared to A/A homozygous patients. Additionally, T/T homozygous patients receiving pomaglumetad methionil had significantly (*p* ≤ 0.05) greater improvements in PANSS total scores compared to placebo and similar improvements as T/T homozygous patients receiving standard-of-care (SOC) treatment. The findings reported here in conjunction with prior reports show that in Caucasian patients with schizophrenia, the T/T genotype at rs7330461 is consistently associated with an increased treatment response to pomaglumetad methionil compared to the A/A genotype.

## 1. Introduction

Schizophrenia has a worldwide lifetime prevalence of 0.5% to 1.6% [1], and patients diagnosed with schizophrenia have a 3.8-fold increased mortality risk compared to the general adult population [2]. Currently, only 25.4% of patients with schizophrenia achieve functional remission worldwide [3]. Additional therapeutic options are needed to improve the prognosis of these patients.

A novel treatment approach for schizophrenia is the modulation of glutamate through interaction with the metabotropic glutamate 2/3 (mGlu2/3) receptors [4]. Pomaglumetad methionil, formerly known as LY2140023 monohydrate and the oral prodrug of LY404039, is a selective agonist for mGlu2/3 receptors [4,5,6]. The efficacy and safety of pomaglumetad methionil for the treatment of schizophrenia has been explored in multiple clinical trials, including HBBD (here, Study 1) [4], HBBR (here, Study 2) [7], HBBM (here, Study 3) [8], HBDE (here, Study 4) [9] and HBBN [10] (here, Study 5; this study was terminated early based on futility analyses). While patients treated with pomaglumetad methionil demonstrated significant improvement in the original proof-of-concept study, Study 1 [4], subsequent studies failed to show efficacy in the general patient population with schizophrenia. Therefore, it was important to identify a subpopulation of patients for whom pomaglumetad methionil would be a viable treatment option. Pharmacogenetic results from the proof-of-concept study identified a significant association between 16 tightly-linked, single nucleotide polymorphisms (SNPs) in the serotonin 2A receptor gene, *HTR2A*, the most significant of which was rs7330461, and response to treatment with pomaglumetad methionil in Caucasian patients with schizophrenia [11]. Caucasian patients carrying the T/T genotype at rs7330461 showed a more favorable response to pomaglumetad methionil compared to patients with either the A/T or A/A genotype at rs7330461 [11]. The validity of this observation was supported by previous studies suggesting potential interactions between 5-HT2A and mGlu2/3 receptors, including 5-HT2A-mGlu2 receptor complexes in the prefrontal cortex that may be involved in the observed crosstalk between these receptor subtypes [12]. Based on these findings, we examined the association of rs7330461 with efficacy outcome in each clinical trial in which pomaglumetad methionil was administered as a monotherapy, deoxyribonucleic acid was collected and a clinical conclusion could be made.

**Table 1:** Study characteristics. SOC, standard-of-care.

Study	Author, Year	Duration (Weeks)	Study Design	Treatments
Study 2	Adams *et al.* 2013 [7]	24 weeks + 28-week extension	Phase 2, randomized, open-label	Pomaglumetad methionil (target: 40 mg BID) SOC (olanzapine (10, 15, or 20 mg, once daily; target = 15 mg/day), aripiprazole (10, 20 or 30 mg, once daily; target = 20 mg/day) or risperidone (2, 4 or 6 mg, once daily or BID; target = 4 mg/day))
Study 3	Downing *et al.* 2014 [8]	6 weeks	Phase 2, randomized, double-blind	Pomaglumetad methionil (40 mg BID or 80 mg BID) Risperidone (2 mg BID) Placebo
Study 4	Adams *et al.* 2014 [9]	24 weeks	Phase 3, randomized, double-blind	Pomaglumetad methionil (flexible dosing: 20 mg to 80 mg BID) Aripiprazole (flexible dosing: 10, 15 or 30 mg)
Study 5	Published on ClinicalTrials.gov., NCT01307800	6 weeks	Phase 3, randomized, double-blind	Pomaglumetad methionil (10 mg BID, 40 mg BID or 80 mg BID) Placebo

Abbreviation: BID = twice daily.

The development plan for pomaglumetad methionil included studies of varying durations in adult patients at different stages over the clinical course of schizophrenia. These studies included: Study 1, a four-week, double-blind, placebo- and olanzapine-controlled proof-of-concept study of pomaglumetad methionil; Study 2, a long-term, open-label safety study comparing pomaglumetad methionil with standard-of-care (SOC) treatment [7]; Study 3, a six-week, double-blind, placebo- and risperidone-controlled study examining the efficacy and safety of pomaglumetad methionil [8]; Study 4, a long-term, double-blind, aripiprazole-controlled study examining the safety and efficacy of pomaglumetad methionil [9]; and Study 5, a six-week double-blind, placebo-controlled study examining the efficacy and safety of pomaglumetad methionil (Table 1) [10]. These individual studies allowed for assessment of rs7330461 within each trial at different time points and across the spectrum of illness. However, efforts to identify and replicate pharmacogenetic markers in the course of drug development face major challenges, including small sample sizes within individual trials and heterogeneous patient populations. To overcome the challenge of small sample sizes in each of the individual studies and to examine the complete evidence across the available studies, we also performed an integrated analysis across four studies, assessing the association between treatment response to pomaglumetad methionil with the *HTR2A* rs7330461 genotype in all Caucasian patients with schizophrenia for whom genetic data were available, as well as in the Caucasian patient subpopulation carrying the rs7330461 T/T genotype. In addition, the association of rs7330461 with response to pomaglumetad methionil was examined in African American patients in Studies 2, 3 and 4.

## 2. Results and Discussion

Presented here are genetic association data from Clinical Studies 2, 3 and 4 individually, as well as integrated analyses of genetic association data from Studies 2, 3, 4 and 5. We demonstrate a genetic association between *HTR2A* SNP rs7330461 and response in Caucasian schizophrenia patients treated with pomaglumetad methionil within multiple clinical trials. Based on this genetic association, we identified a subpopulation of Caucasian patients who show improved response to pomaglumetad methionil *versus* placebo. This is in contrast to the general patient population, in which a significant treatment response has not been observed in patients treated with pomaglumetad methionil compared to placebo [8]. To our knowledge, this is the first pharmacogenetic study to identify and replicate in multiple studies a genetic marker of efficacy for the treatment of schizophrenia that does not rely on drug metabolism genes.

Genetic data from Study 1 were not included in the integrated analysis, because Study 1 was the only clinical trial in which pomaglumetad methionil showed clinical efficacy relative to placebo in the overall population. However, despite the exclusion of the initial genetic association results (Study 1), the pharmacogenetic signal remained significant in the integrated analysis.

### 2.1. Patient Baseline Demographics and Illness Characteristics in Studies 2 through 5

Among Caucasian patients, pharmacogenetic data were available for a total of 1115 patients, including 513 A/A homozygous, 466 A/T heterozygous and 136 T/T homozygous patients across Studies 2 to 5 (Table 2). Overall, no differences in baseline demographics and illness characteristics were observed among the three rs7330461 genotype groups, with the exception of T/T homozygous patients in Study 3, who were significantly younger (*p* = 0.016) and more frequently male (*p* = 0.023) compared to A/A homozygous or A/T heterozygous patients (Table 1).

Among African American patients, pharmacogenetic data were available for a total of 696 patients, including 137 A/A homozygous patients, 333 A/T carriers and 226 T/T homozygous patients across Studies 2, 3 and 4 (Table 2). No differences in baseline demographics and illness characteristics were observed among rs7330461 genotypes (Table 2).

**Table 2:** Baseline demographics and efficacy measures across rs7330461 genotypes.

Study		A/A	A/T	T/T	Overall *p*-Value
Study 2	Caucasian patients	N = 51	N = 48	N = 17	
	Age, mean (SD) years	38.2 (11.8)	41.5 (12.7)	38.1 (10.6)	0.344 ^1^
	Sex, male, n (%)	32 (62.7)	26 (54.2)	13 (76.5)	0.274 ^2^
	PANSS total score, mean (SD)	85.3 (11.5)	81.9 (12.2)	79.5 (11.3)	0.154 ^1^
	African American patients	N = 20	N = 34	N = 25	
	Age, mean (SD) years	44.9 (10.0)	41.8 (11.9)	41.0 (11.7)	0.490 ^1^
	Sex, male, n (%)	17 (85.0)	28 (82.4)	19 (76.0)	0.814 ^2^
	PANSS total score, mean (SD)	88.2 (11.4)	83.5 (11.8)	81.5 (14.5)	0.206 ^1^
Study 3	Caucasian patients	N = 265	N = 215	N = 69	
	Age, mean (SD), years	39.0 (11.9)	41.3 (12.3)	36.9 (11.2)	0.016 ^1^
	Sex, male, n (%)	152 (57.4)	116 (54.0)	50 (72.5)	0.023 ^2^
	PANSS total score, mean (SD)	84.3 (14.7)	84.1 (13.9)	88.0 (20.5)	0.148 ^1^
	African American patients	N = 64	N = 146	N = 96	
	Age, mean (SD) years	41.9 (11.5)	40.3 (11.1)	40.8 (10.5)	0.606 ^1^
	Sex, male, n (%)	48 (75.0)	105 (71.9)	67 (69.8)	0.782 ^2^
	PANSS total score, mean (SD)	84.7 (13.6)	82.1 (13.2)	81.7 (13.5)	0.328 ^1^
Study 4	Caucasian patients	N = 112	N = 126	N = 30	
	Age, mean (SD), years	42.2 (12.1)	43.0 (11.7)	42.3 (12.1)	0.881 ^1^
	Sex, male, n (%)	63 (56.3)	83 (65.9)	19 (63.3)	0.303 ^2^
	PANSS total score, mean (SD)	83.1 (29.0)	83.5 (32.9)	84.0 (28.5)	0.990 ^1^
	African American patients	N = 53	N = 153	N = 96	
	Age, mean (SD), years	44.1 (9.1)	42.5 (10.0)	43.4 (10.4)	0.537 ^1^
	Sex, male, n (%)	33 (62.3)	100 (65.4)	63 (65.6)	0.909 ^2^
	PANSS total score, mean (SD)	69.3 (16.5)	74.5 (15.9)	75.5 (15.3)	0.060 ^1^
Study 5	Caucasian patients	N = 85	N = 77	N = 20	
	Age, mean (SD), years	40.5 (10.9)	41.5 (11.5)	42.4 (10.1)	0.751 ^1^
	Sex, male, n (%)	59 (69.4)	53 (68.8)	12 (60.0)	0.705 ^2^
	PANSS total score, mean (SD)	80.8 (14.7)	82.0 (13.6)	80.6 (9.1)	0.824 ^1^
	African American patients	N = 57	N = 118	N = 89	
	Age, mean (SD), years	39.9 (11.1)	42.7 (10.5)	41.4 (11.3)	0.275 ^1^
	Sex, male, n (%)	42 (73.7)	82 (69.5)	68 (76.4)	0.557 ^2^
	PANSS total score, mean (SD)	81.4 (15.3)	82.1 (12.7)	81.7 (13.4)	0.938 ^1^

^1^ *p*-values are from the Type 3 sums of squares ANOVA model with genotype as an independent factor. ^2^ *p*-values are from a Fisher exact test. Abbreviations: ANOVA = analysis of variance; N = total number of patients; n = number of patients in a specified category; PANSS = Positive and Negative Symptom Scale; SD = standard deviation.

### 2.2. Effect of SNP rs7330461 Genotype in Studies 1, 2, 3 and 4

**Figure 1:**
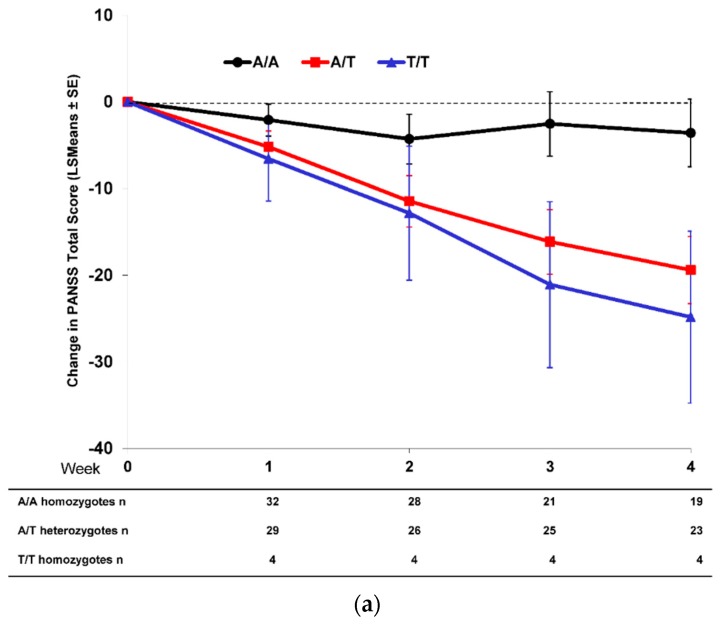

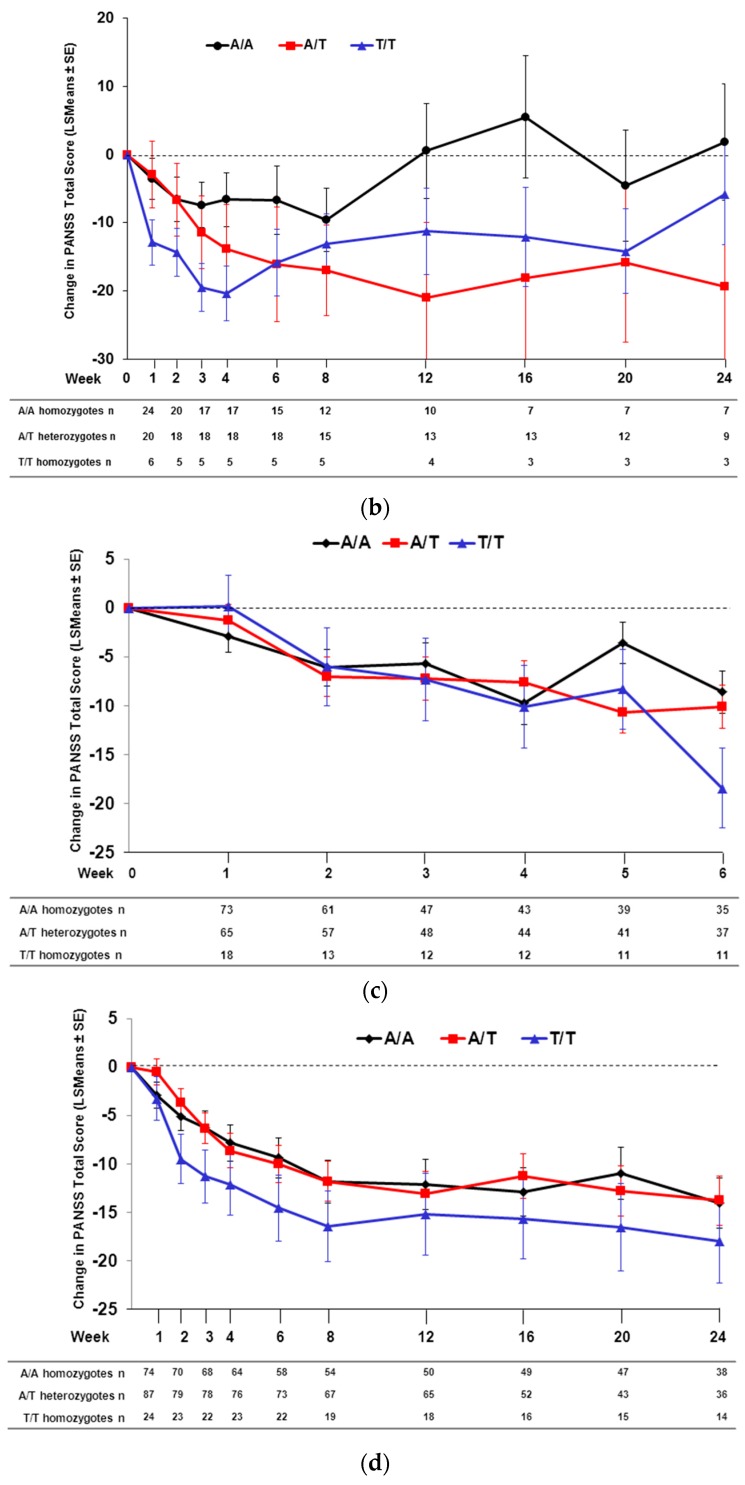
Change in PANSS total score in Caucasian patients treated with pomaglumetad methionil 40 mg BID by *HTR2A* SNP rs7330461 genotype. (**a**) Study 1. (**b**) Study 2. (**c**) Study 3. (**d**) Study 4. Abbreviations: BID = twice daily; *HTR2A* = serotonin 2A receptor (*HTR2A*) gene; n = number of patients with PANSS measures; LSMeans = least squares means; PANSS = Positive and Negative Syndrome Scale; SE = standard error; SNP = single nucleotide polymorphism.

As previously published [11], Caucasian patients homozygous for the T/T genotype at SNP rs7330461 showed the greatest improvement in Positive and Negative Syndrome Scale (PANSS) total score during treatment with pomaglumetad methionil 40 mg twice daily (BID) in Study 1 compared to A/T and A/A genotypes (Figure 1a). Similar results were observed in Studies 2, 3 and 4, with patients homozygous for the T/T genotype at SNP rs7330461 showing greater treatment response to pomaglumetad methionil 40 mg BID as assessed with the change in PANSS total score compared to patients homozygous for the A/A genotype (Figure 1b–d). In Study 3, patients homozygous for the T/T genotype experienced greater treatment response to pomaglumetad methionil 40 mg BID compared to patients homozygous for the A/A genotype only at Week 6.

**Figure 2:**
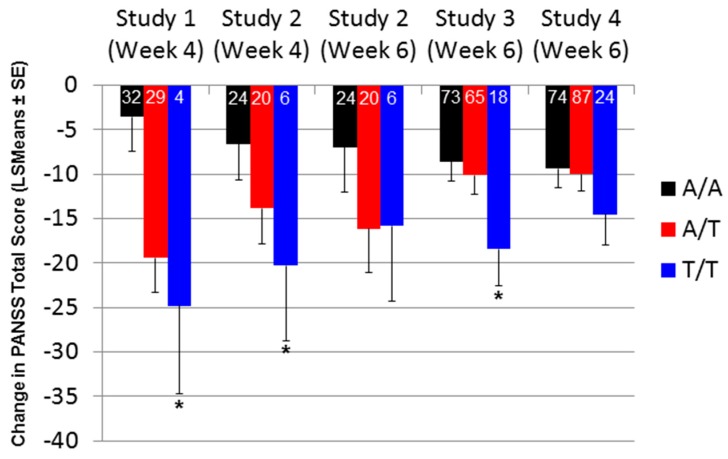
Change in PANSS total score by the *HTR2A* SNP rs7330461 genotype in Caucasian patients treated with pomaglumetad methionil 40 mg BID in four different studies. Abbreviations: BID = twice daily; *HTR2A* = serotonin 2A receptor (*HTR2A*) gene; LSMeans = least squares means; PANSS = Positive and Negative Syndrome Scale; SE = standard error; SNP = single nucleotide polymorphism. Numbers in the bars indicate the number of patients with PANSS measures included in the analyses. * *p* ≤ 0.05, two-sided *p*-value, testing is based on comparing A/A *versus* A/T *versus* T/T for Study 1; one-sided *p-*value for Studies 2, 3 and 4.

**Figure 3:**
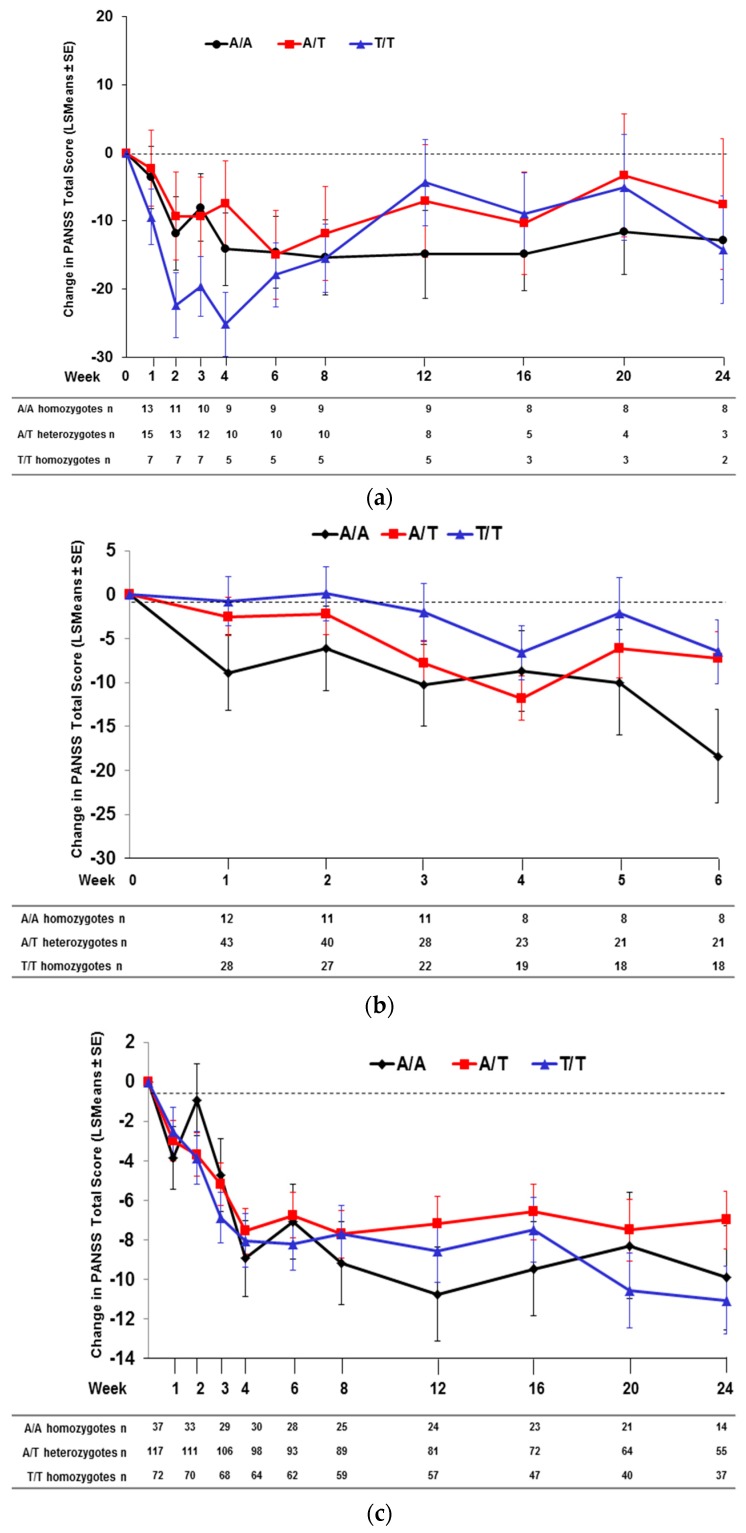
Change in PANSS total score in African American patients treated with pomaglumetad methionil 40 mg BID by *HTR2A* SNP rs7330461 genotype. (**a**) Study 2. (**b**) Study 3. (**c**) Study 4. Abbreviations: BID = twice daily; n = number of patients with PANSS measures; *HTR2A* = serotonin 2A receptor (*HTR2A*) gene; LSMeans = least squares means; PANSS = Positive and Negative Syndrome Scale; SE = standard error; SNP = single nucleotide polymorphism.

**Figure 4:**
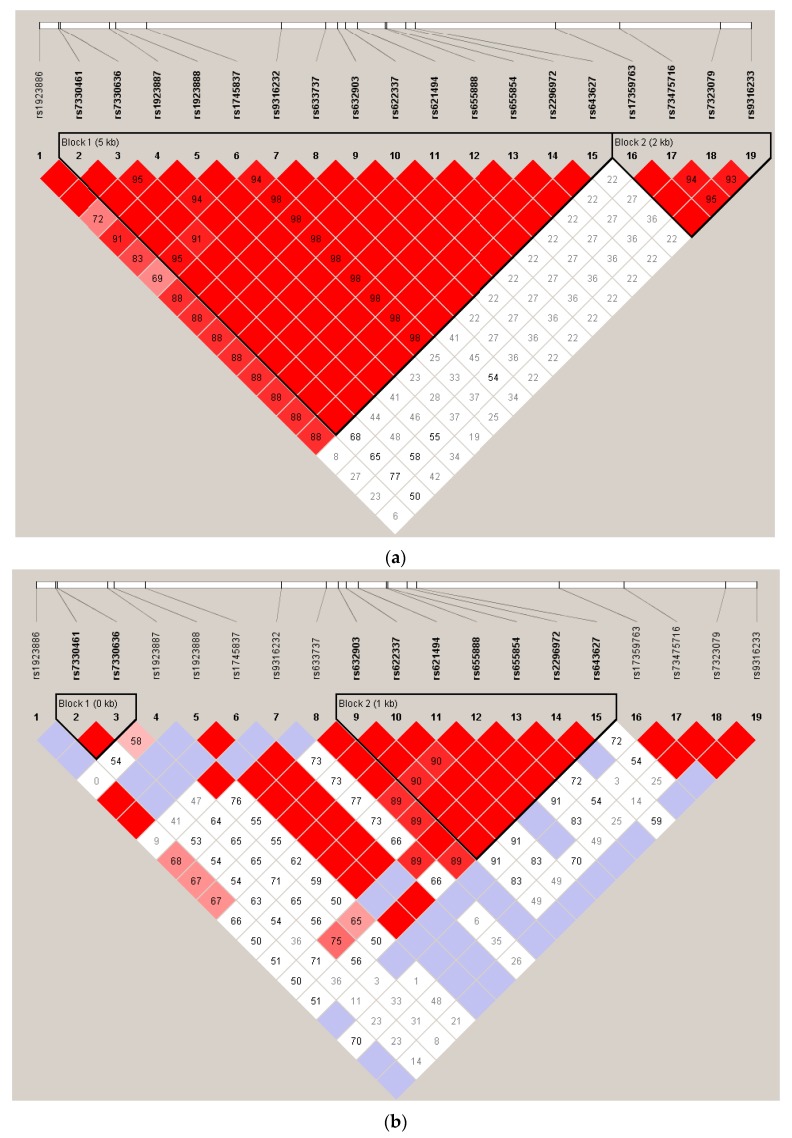
Linkage disequilibrium plots of rs733046 in *HTR2A* in Caucasian and African American patients. (**a**) Caucasian patients. (**b**) African American patients. The linkage disequilibrium (LD) plots were created using Haploview, and the color code on the plot follows the standard color scheme for Haploview: white = (|D′| < 1, logarithm (base 10) of odds (LOD) < 2), shades of pink/red = (|D′| < 1, LOD ≥ 2), blue = (|D′| = 1, LOD < 2), bright red = (|D′| = 1, LOD ≥ 2). Numbers on top of the plot are the reference sequence ID (rs-number) of the included single nucleotide polymorphisms (SNPs) with the distance between them indicated on the white bar above.

When comparing PANSS total score changes after 4 and/or 6 weeks of treatment with pomaglumetad methionil 40 mg BID among genotypes across the individual analyses conducted in the four studies, Caucasian patients homozygous for the T/T genotype at SNP rs7330461 consistently showed greater score improvements compared to patients homozygous for the A/A genotype (Figure 2). The differences between T/T and A/A genotypes reached statistical significance (*p* ≤ 0.05) in Studies 1 and 2 at Week 4 and in Study 3 at Week 6. Our initial pharmacogenetic analysis was limited to Caucasian individuals because the vast majority of patients (>99%) in Study 1 were from this racial group. Subsequent studies included enrollment of African American patients and allowed for an assessment of the genetic association between SNP rs7330461 and treatment response to pomaglumetad methionil in this racial group. The effect of SNP rs7330461 genotype on response to treatment with pomaglumetad methionil in African American patients as assessed by the change in PANSS total score was examined in Studies 2, 3 and 4 (Figure 3a–c). In contrast to Caucasian patients, no consistent effect of SNP rs7330461 genotype was observed in African American patients. Linkage disequilibrium (LD) plots were generated for the *HTR2A* gene in Caucasian and African American populations using Haploview (Figure 4a,b). Examination of the region including SNP rs7330461 revealed very different patterns of LD in this area of the gene, with the LD block observed in Caucasian patients limited to two SNPs in African American patients (Figure 4). These results are limited by the patients’ self-identification of race, which might cause misclassifications. While no significant deviations from Hardy–Weinberg equilibrium were observed, historically it is acknowledged that racial self-identification can be problematic in clinical studies.

It is also possible that other genetic variants, through linkage or interaction, may be responsible for the detected association with rs7330461. To examine this possibility, we sequenced the *HTR2A* gene from Caucasian and African American patients enrolled in Studies 1 and 2. However, no SNPs more strongly associated with treatment response than rs7330461 could be identified. Given these findings, as well as the differences in LD structure between Caucasians and African Americans for the *HTR2A* gene (Figure 4), these data suggest that SNP rs7330461 is tagging a yet-to-be-identified causal variant responsible for the differential treatment response to pomaglumetad methionil in Caucasian patients.

### 2.3. Treatment Response in Caucasian Patients Homozygous for the T/T Genotype in Study 3

**Figure 5:**
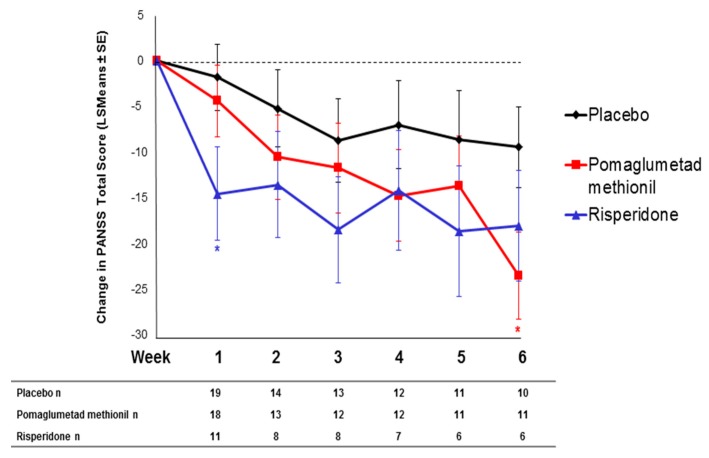
Change in PANSS total score in Caucasian T/T homozygous patients treated with pomaglumetad methionil 40 mg BID, risperidone 2 mg/day or placebo. Abbreviations: BID = twice daily; n = number of patients with PANSS measures; LSMeans = least squares means; PANSS = Positive and Negative Syndrome Scale; SE = standard error. * *p* ≤ 0.05, two-sided *p*-value.

To examine the response to pomaglumetad methionil treatment *versus* placebo in Caucasian patients homozygous for the T/T genotype, change in PANSS total score was examined in Study 3, because this study had the largest population of treated patients within a single clinical trial. Caucasian T/T homozygous patients treated with pomaglumetad methionil showed a significantly greater decrease in PANSS total score at Week 6 (two-sided *p* = 0.0367) compared to T/T homozygous patients treated with placebo (Figure 5). A significant treatment effect between risperidone and placebo in the PANSS total score change was observed in the T/T group at Week 1 (two-sided *p* = 0.0325), but not at Week 6 (two-sided *p* = 0.2474).

### 2.4. Effect of SNP rs7330461 Genotype in the Integrated Analysis

Data were pooled from Studies 2, 3, 4 and 5 (Study 5 was only included in integrated analysis, due to the small sample size) to analyze the mean change in PANSS total scores from baseline through Week 6 of treatment with pomaglumetad methionil 40 mg BID by the rs7330461 genotype. Data from Study 1 were not included in this analysis, because this study was only four weeks in duration, and in Study 1, patients treated with pomaglumetad methionil within the overall population showed significantly greater improvements in PANSS total score compared to placebo patients, while in the subsequent clinical trials, this separation was not observed.

**Figure 6:**
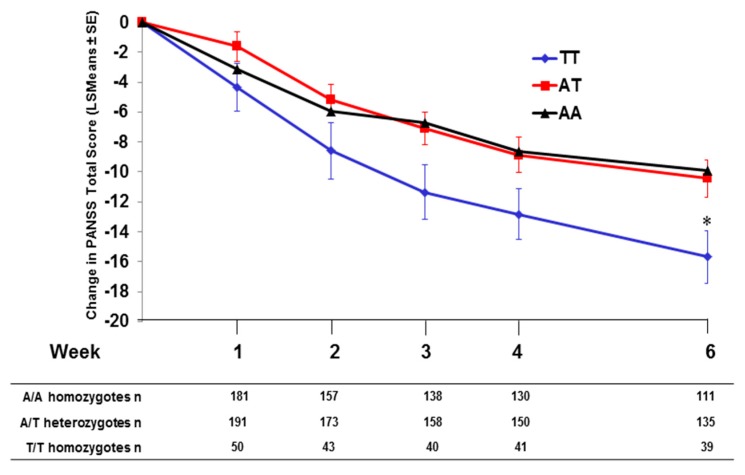
Integrated analysis; change in PANSS total score by *HTR2A* SNP rs7330461 genotype in Caucasian patients treated with pomaglumetad methionil 40 mg BID; data from Studies 2, 3, 4 and 5. Abbreviations: BID = twice daily; *HTR2A* = serotonin 2A receptor (*HTR2A*) gene; LSMean = least squares mean; n = number of patients with PANSS measures; PANSS = Positive and Negative Syndrome Scale; SE = standard error; SNP = single nucleotide polymorphism. Type I error rate < 0.05 for T/T being the most responsive genotype based on Hsu’s multiple comparisons with the best (MCB) method in the PANSS total analysis. * Two-sided *p* = 0.0073 for T/T *versus* A/A and two-sided *p* = 0.0098 for T/T *versus* A/T.

In the integrated analysis, patients in the rs7330461 T/T genotype group treated with pomaglumetad methionil 40 mg BID were the most responsive based on Hsu’s multiple comparisons with the best (MCB) method (Type 1 error rate < 0.05). Patients who were T/T homozygous showed a significantly (*p* = 0.0073) greater improvement in PANSS total score compared to A/A homozygous patients at Week 6 of treatment (Figure 6).

### 2.5. Pomaglumetad Methionil versus Placebo in the Integrated Analysis

**Figure 7:**
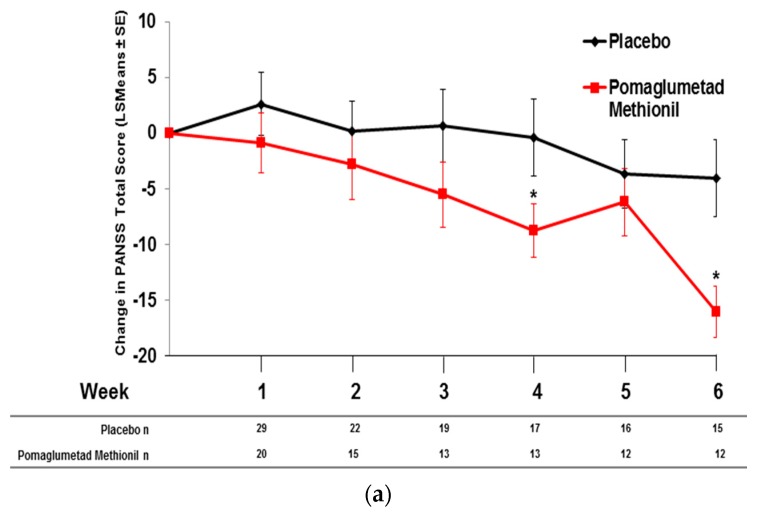

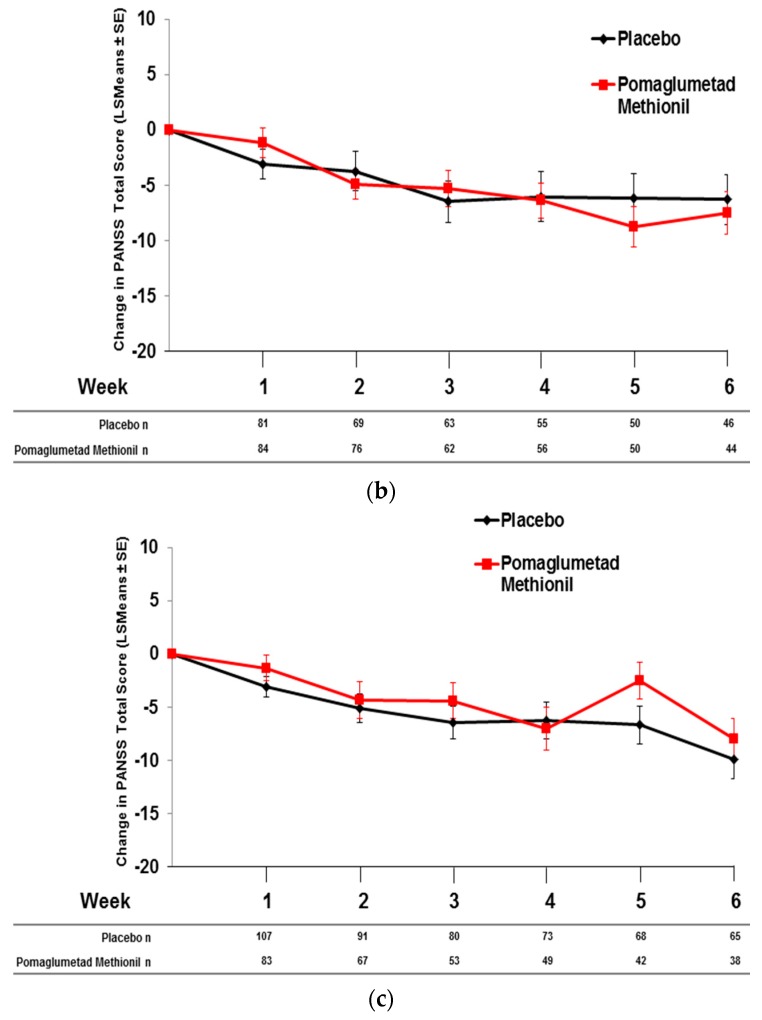
Integrated analysis; change in PANSS total score by *HTR2A* SNP rs7330461 genotype in Caucasian patients treated with pomaglumetad methionil *versus* placebo; data from Studies 3 and 5. (**a**) T/T homozygous patients. (**b**) A/T heterozygous patients. (**c**) A/A homozygous patients. Abbreviations: *HTR2A* = serotonin 2A receptor (*HTR2A*) gene; LSMeans = least squares means; n = number of patients with PANSS measures; PANSS = Positive and Negative Syndrome Scale; SE = standard error; SNP = single nucleoside polymorphism.

To examine the effect of rs7330461 genotype on mean change from baseline in PANSS total score during treatment with pomaglumetad methionil 40 mg BID *versus* placebo, data from studies including a placebo arm (Studies 3 and 5) were pooled. Patients homozygous for T/T showed a significantly (*p* ≤ 0.049) greater improvement in mean PANSS total score during treatment with pomaglumetad methionil compared to placebo at Weeks 4 and 6 (Figure 7a). No significant differences between pomaglumetad methionil and placebo treatment groups were observed in A/T heterozygous (Figure 7b) or A/A homozygous patients (Figure 7c).

### 2.6. Pomaglumetad Methionil versus Standard-Of-Care in the Integrated Analysis

**Figure 8:**
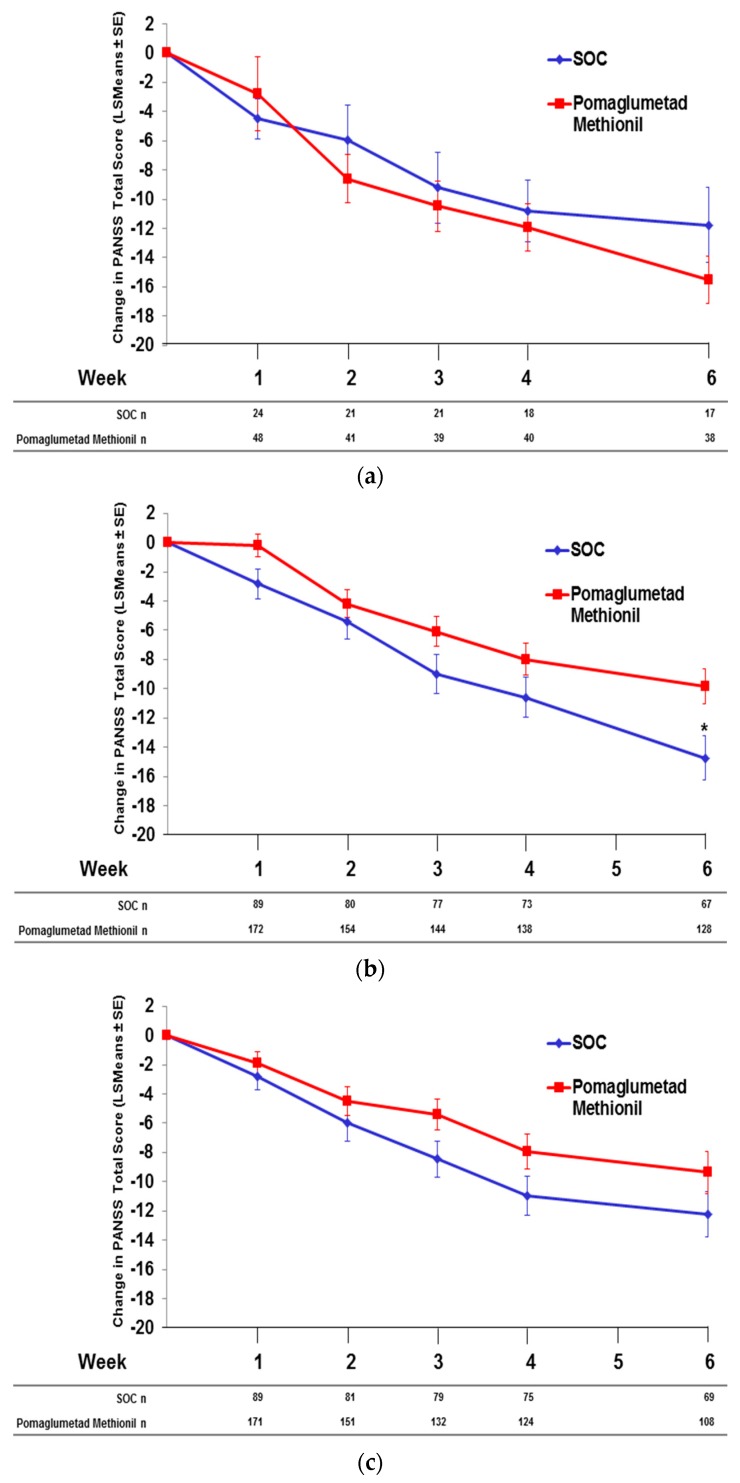
Integrated analysis; change in PANSS total score by *HTR2A* SNP rs7330461 genotype in Caucasian patients treated with pomaglumetad methionil *versus* standard of care; data from Studies 2, 3 and 4. (**a**) T/T homozygous patients. (**b**) A/T heterozygous patients. (**c**) A/A homozygous patients. Abbreviations: *HTR2A* = serotonin 2A receptor (*HTR2A*) gene; LSMeans = least squares means; n = number of patients with PANSS measures; PANSS = Positive and Negative Syndrome Scale; SE = standard error; SOC = standard of care; SNP = single nucleoside polymorphism.

To examine the effect of rs7330461 genotype on mean change from baseline in PANSS total score during treatment with pomaglumetad methionil 40 mg BID *versus* SOC, data from studies including an SOC treatment arm (Studies 2, 3 and 4) were pooled. Patients homozygous for T/T showed similar improvements in mean PANSS total score during treatment with pomaglumetad methionil compared to SOC over six weeks of treatment (Figure 8a). In A/T heterozygous patients, SOC treatment resulted in a significantly (*p* = 0.009) greater improvement of mean PANSS total score compared to pomaglumetad methionil (Figure 8b). No significant differences between pomaglumetad methionil and SOC treatment groups were observed in A/A homozygous patients (Figure 8c). In a recent *post hoc* analysis, patients previously treated with atypical antipsychotics did not respond to pomaglumetad methionil, whereas those previously treated with typical antipsychotics did respond to pomaglumetad methionil. However, treatment with risperidone did not appear to depend on prior treatment with typical or atypical antipsychotics [13].

### 2.7. Potential Functional Effect of HTR2A SNP 7330461

The SNP rs7330461 is located within the large intron of the *HTR2A* gene [11]. To date, there is no evidence that this SNP, or any in LD with this SNP, is associated with schizophrenia disease risk as assessed in the May 2013 freeze of the Psychiatric Genomics Consortium dataset [14], using the Ricopili tool [15]. Evidence from ENCODE (Encyclopedia of DNA Elements) suggests that rs7330461 (or an SNP in LD) may affect chromatin structure and subsequently influence HTR2A expression [16]. Specifically, there are three potential regulatory features near rs7330461 (chr13: 47423815). The first is 1215 bp upstream and is based on ChIP-seq data. This feature predicts a repressed or low activity region at position chr13:47422201-47422600. The second is 606 bp downstream and represents a DNase cluster at position chr13:47424421-47424670. DNase hypersensitive areas indicate regulatory regions and promoters. The third is 668 bp downstream and is based on ChIP-seq data. This predicts a weak enhancer or open chromatin cis regulatory element at position chr13:47424483-47424713 [16]. While a functional effect for rs7330461 has yet to be demonstrated experimentally, its location within the region of a putative antisense nested gene suggests a potential role for the polymorphism in regulating this antisense nested gene or the *HTR2A* gene itself [11]. Previously, it has been suggested that *HTR2A* expression is needed for antipsychotic effects of mGlu2/3 agonists [17]. In patients homozygous for T/T at rs7330461, there may conceivably be a 5HT2A-mediated inability of histone deacetylases to bind to the mGlu2 promoter, thus allowing sufficient histone acetylation for adequate transcription of mGlu2 and receptor activation by pomaglumetad [18]. Crosstalk between 5-HT2A and mGlu2/3 receptors has been demonstrated with mGlu2/3 receptor knockout mice, in which the pharmacological and behavioral effects of hallucinogenic 5-HT2A agonists are abolished [19]. Further evidence for crosstalk between both receptors lies in the fact that in 5-HT2A knockout mice, an attenuation of the locomotor activity induced by the mGlu2/3 receptor antagonist LY341495 is observed [20]. In mouse and human cortical pyramidal neurons, the mGlu2 and 5-HT2A receptors form a heterocomplex, which results in potentiation of glutamate-induced mGlu2 signaling and attenuation of serotonin-induced 5-HT2A signaling compared to homomeric responses [20]. Thus, altered expression of the *HTR2A* gene in patients homozygous for T/T at rs7330461 could potentially lead to enhanced treatment response to pomaglumetad methionil.

### 2.8. Integration of Pharmacogenetic Analyses during Clinical Drug Development

In addition to unique association results for SNP rs7330461, the pharmacogenetic analysis of data from the pomaglumetad methionil clinical development program provides critical learning about the challenges of integrating genetic analysis into drug development. Tension exists between the development plan needs for drug registration (e.g., geographical diversity, minority recruitment, speed to the patient, required exposures, balanced randomization) and the ideal setting for identifying and replicating markers of treatment response to a novel therapeutic. The assessment of a consistent pharmacogenetic effect in a drug development program without a consistent overall clinical response or known function of the variant introduces additional challenges even beyond those typically present. With the emerging data around the complexities of schizophrenia disease genetics independent of treatment response [14], it is likely that meaningfully-sized subpopulations would need to be identified through multiple genetic factors.

## 3. Experimental Section

Before patient enrollment, the appropriate institutional review boards reviewed and approved the study protocols. All patients provided written informed consent before receiving any study therapy or undergoing any study procedure, and the studies were performed according to Good Clinical Practice guidelines. To allow assessment of a genetically homogeneous population, the initial analyses were limited to self-identified non-Hispanic white patients, herein referred to as Caucasians. The combined use of race and ethnicity information for patient inclusion in the pharmacogenetic analysis is based on the United States Department of Health and Human Services guidance on data collection standards, which were used in the clinical trials [21].

### 3.1. Clinical Trial Designs and Patients

Detailed descriptions of trial designs and inclusion/exclusion criteria are available at ClinicalTrials.gov under NCT00845026 (Study HBBR = Study 2), NCT01086748 (Study HBBM = Study 3), NCT01328093 (Study HBDE = Study 4) and NCT01307800 (Study HBBN = Study 5). Study 2 was a phase 2, randomized, open-label, active comparator-controlled, 24-week (plus 28-week extension) clinical trial examining the safety of pomaglumetad methionil (target dose: 40 mg BID) *versus* SOC treatment in adult patients with schizophrenia as defined by the Diagnostic and Statistical Manual of Mental Disorders, 4th edition, text revision (DSM-IV-TR) [7]. Study 3 was a phase 2, randomized, double-blind, placebo- and risperidone-controlled, 6-week clinical trial examining the efficacy and safety of pomaglumetad methionil (40 mg and 80 mg BID) in adult patients with schizophrenia as defined by the DSM-IV-TR [8]. Analyses were performed for all randomized intent-to-treat (ITT) Caucasian patients, with genotype data, who were not identified as placebo responders (≥25% improvement on PANSS total scores) during the blinded lead-in phase. Study 4 was a phase 3, randomized, double-blind, aripiprazole-controlled (flexibly dosed: 10, 15 or 30 mg/day), 24-week clinical trial examining the safety and efficacy of pomaglumetad methionil (flexibly dosed between 20 mg and 80 mg BID) in adult (18 to 65 years of age) patients with schizophrenia according to DSM-IV-TR criteria [9]. Study 5 was a phase 3, randomized, double-blind, placebo-controlled, 6-week study examining the efficacy and safety of pomaglumetad methionil (fixed doses of 80 mg BID, 40 mg BID or 10 mg BID) in adult patients (18 to 65 years old) with DSM-IV-TR schizophrenia (Table 1). Due to small sample size and early termination of Study 5, genetic data from this study were not analyzed individually and were only included in the integrated analysis.

### 3.2. Genotyping

Multiple sets of SNPs were genotyped as part of the individual studies. In the current analyses, we focus primarily on the genetic association between SNP rs7330461 in *HTR2A* and response to pomaglumetad methionil 40 mg BID in Caucasians based on results from a prior study showing a significant association of this genetic variant with efficacy of pomaglumetad methionil in Caucasian patients with schizophrenia at the 40-mg BID dose (unadjusted *p* = 0.0005) [11] and because the number of patients treated with other doses was too few to warrant genetic analysis. Genotyping was performed by Genizon BioSciences, Inc. (St. Laurent, QC, Canada), using Illumina’s GoldenGate platform (Illumina, San Diego, CA, USA; Study 2), by Covance Central Laboratories Services (Indianapolis, IN, USA) using Verigene® mGlu Nucleic Acid Test (Nanosphere, Chicago, IL, USA; Study 3) and TaqMan (Life Technologies, Grand Island, NY, USA; Studies 4 and 5).

### 3.3. Genotype Data Quality Control

To ensure the integrity of the data for the genetic analyses, the genotype data quality was examined based on the total number of SNPs that were genotyped in the individual studies. The percentages of patients with usable rs7330461 genotype data and Hardy–Weinberg equilibrium within each trial were examined (Table 3).

**Table 3:** Genotyping data characteristics.

Study	Patients with rs7330461 Genotype, n/N (%)	HWE for rs7330461
2	253/261 (96.9)	0.303
3	1011/1013 (99.8)	0.105
4	676/678 (99.7)	0.591
5	559/567 (98.6)	0.513

Abbreviations: HWE = Hardy–Weinberg equilibrium; n = number of patients with genotype data; N = total number of patients.

### 3.4. Statistical Analyses

Change from baseline in the PANSS total score for Caucasian patients treated with pomaglumetad methionil was assessed in each clinical study and used as the primary efficacy measure for all pharmacogenetic analyses. Deviations from Hardy–Weinberg equilibrium were obtained using an exact test. Associations between *HTR2A* SNP rs7330461 and changes in PANSS total score were analyzed with a mixed-model repeated measures (MMRM) analysis under a genotypic assumption (*i.e.*, genotype was treated as a categorical variable). The MMRM model for all studies included the baseline PANSS total score and baseline score-by-visit interaction as covariates and country, sex, genotype, visit and genotype-by-visit interaction as fixed effects. The MMRM model for Study 3 within the rs7330461 T/T genotype included baseline PANSS total score and baseline score-by-visit interaction as covariates and investigative site, sex, treatment, visit and treatment-by-visit interaction as fixed effects. The same models were run within African American patients for Studies 2, 3 and 4.

#### Integrated Analyses

To further investigate the association of rs7330461 with response to treatment with pomaglumetad methionil in Caucasian patients, an integrated analysis combining data from the individual studies was conducted to assess the consistency of association and to compare the treatment effect of pomaglumetad methionil with placebo and with SOC in the T/T genotype population. Only Caucasian patients treated with pomaglumetad methionil 40 mg BID, placebo or SOC were included in the analyses, and the treatment duration was limited to 6 weeks. The change from baseline in the PANSS total score was assessed for T/T, A/T and A/A genotype groups of the *HTR2A* SNP rs7330461 in Caucasian patients using MMRM analysis over 6 weeks. The MMRM model included baseline PANSS total score and baseline-by-visit as covariates and visit, study, country (nested in study), genotype and genotype-by-visit as fixed effects. Hsu’s MCB method was applied for multiplicity adjustment to identify the most responsive genotype group [22]. Treatment effect (pomaglumetad methionil *versus* placebo and pomaglumetad methionil *versus* SOC) within each genotype was also assessed based on MMRM analyses over 6 weeks. Studies 3 and 5 were used to make comparisons between pomaglumetad methionil and placebo; Studies 2, 3 and 4 were used for comparisons between pomaglumetad methionil and SOC. The MMRM model included baseline PANSS total score and baseline-by-visit as covariates and study, country (nested in study), treatment, visit, treatment-by-visit, study-by-visit, study-by-treatment, genotype, genotype-by-visit, genotype-by-treatment and genotype-by-treatment-by-visit as fixed effects.

## 4. Conclusions

In conclusion, in Caucasian patients with schizophrenia, the T/T genotype at rs7330461 has consistently been shown to be associated with an increased treatment response to pomaglumetad methionil compared to the A/A genotype, implicating the potential involvement of *HTR2A* genetic variants including rs7330461 in the treatment response to glutamatergic agents. The findings presented here might aid in the understanding of the functional relevance of this gene region and are supportive of prior findings in rodent models elucidating the effects of selective mGlu2/3 receptor agonists on HTR2A [18]. Further studies in this genotype group with pomaglumetad methionil or with compounds with a similar mechanism of action are needed to gain more understanding of the significance of this finding. Nevertheless, these findings may open potential avenues for research on glutamatergic mechanisms in schizophrenia and their relationship with serotonergic systems.
